# Supplementary material for: Deletion of wfs1 Impairs Oligodendrocyte Precursor Cells Dorsal Distribution and Myelination Through the wfs1-hmgcs1 Axis in Zebrafish
Source: Biology (Basel). 2026 Aug 16;15(16):1402. doi: 10.3390/biology15161402 (PMC13510151; doi:10.3390/biology15161402)
Supplement: Supplementary file 1 [file biology-15-01402-s001.zip › File S1-Transcriptome sequencing data and differentially expressed genes (DEGs) in wfs1-- zebrafish at 5 dpf/1.GO/wfs1KOvsWT/GSEA/wfs1KO/SNAP_RECEPTOR_ACTIVITY/SNAP_RECEPTOR_ACTIVITY(GO_0005484).html]

Details for gene set SNAP\_RECEPTOR\_ACTIVITY(GO:0005484)[GSEA]


|  || Dataset | deg.sample |
| Phenotype | sample.cls |
| Upregulated in class | wfs1KO |
| GeneSet | SNAP\_RECEPTOR\_ACTIVITY(GO:0005484) |
| Enrichment Score (ES) | 0.53064984 |
| Normalized Enrichment Score (NES) | 1.8988916 |
| Nominal p-value | 0.0 |
| FDR q-value | 0.077373154 |
| FWER p-Value | 0.34 |
Table: GSEA Results Summary

  

Fig 1: Enrichment plot: SNAP\_RECEPTOR\_ACTIVITY(GO:0005484)
  
Profile of the Running ES Score & Positions of GeneSet Members on the Rank Ordered List

  

| PROBE | DESCRIPTION   (from dataset) | GENE SYMBOL | GENE\_TITLE | RANK IN GENE LIST | RANK METRIC SCORE | RUNNING ES | CORE ENRICHMENT || 1 | 436605 | stx5a |  |  | 32 | 4.929 | 0.1489 | Yes |
| 2 | 449923 | si:ch73-234b20.5 |  |  | 327 | 3.546 | 0.2475 | Yes |
| 3 | 571872 | zgc:165520 |  |  | 964 | 2.142 | 0.2926 | Yes |
| 4 | 393236 | sybl1 |  |  | 967 | 2.136 | 0.3575 | Yes |
| 5 | 393836 | stx11b.1 |  |  | 1443 | 1.695 | 0.3941 | Yes |
| 6 | 325484 | vamp5 |  |  | 1642 | 1.574 | 0.4357 | Yes |
| 7 | 445167 | stx2a |  |  | 1898 | 1.414 | 0.4707 | Yes |
| 8 | 327007 | vamp8 |  |  | 2179 | 1.294 | 0.5013 | Yes |
| 9 | 393346 | stx8 |  |  | 2411 | 1.204 | 0.5306 | Yes |
| 10 | 492808 | stx17 |  |  | 3434 | 0.945 | 0.5273 | No |
| 11 | 415135 | bet1 |  |  | 6204 | 0.568 | 0.4576 | No |
| 12 | 100006625 | stx1a |  |  | 6986 | 0.493 | 0.4481 | No |
| 13 | 795732 | bnip1b |  |  | 9195 | 0.353 | 0.3895 | No |
| 14 | 569124 | stx12l |  |  | 9356 | 0.344 | 0.3949 | No |
| 15 | 751727 | snap47 |  |  | 9894 | 0.310 | 0.3874 | No |
| 16 | 393159 | snap23.1 |  |  | 10097 | 0.296 | 0.3901 | No |
| 17 | 562857 | stx16 |  |  | 12041 | 0.188 | 0.3348 | No |
| 18 | 393515 | stx3a |  |  | 12741 | 0.152 | 0.3175 | No |
| 19 | 114464 | sec22ba |  |  | 12870 | 0.147 | 0.3179 | No |
| 20 | 323735 | stx4 |  |  | 13648 | 0.109 | 0.2969 | No |
| 21 | 553460 | snap29 |  |  | 14882 | 0.052 | 0.2597 | No |
| 22 | 30712 | snap25a |  |  | 14927 | 0.050 | 0.2599 | No |
| 23 | 562669 | stx7l |  |  | 16112 | -0.006 | 0.2228 | No |
| 24 | 415141 | stx12 |  |  | 16442 | -0.016 | 0.2130 | No |
| 25 | 323105 | stx5al |  |  | 16849 | -0.028 | 0.2011 | No |
| 26 | 30711 | snap25b |  |  | 17054 | -0.035 | 0.1957 | No |
| 27 | 445493 | bet1l |  |  | 17624 | -0.059 | 0.1797 | No |
| 28 | 436805 | zgc:92912 |  |  | 17804 | -0.068 | 0.1761 | No |
| 29 | 406707 | vamp1 |  |  | 19580 | -0.147 | 0.1249 | No |
| 30 | 336281 | vamp2 |  |  | 19711 | -0.153 | 0.1254 | No |
| 31 | 415163 | vamp3 |  |  | 20257 | -0.177 | 0.1137 | No |
| 32 | 58038 | stx1b |  |  | 22861 | -0.317 | 0.0416 | No |
| 33 | 554130 | stx18 |  |  | 24476 | -0.418 | 0.0036 | No |
| 34 | 324569 | gosr2 |  |  | 24481 | -0.419 | 0.0162 | No |
| 35 | 447892 | zgc:101731 |  |  | 25359 | -0.472 | 0.0031 | No |
| 36 | 101882035 | si:ch211-208k15.1 |  |  | 25607 | -0.494 | 0.0103 | No |
| 37 | 100005065 | stx11b.2 |  |  | 25648 | -0.498 | 0.0242 | No |
| 38 | 641321 | snap23.2 |  |  | 25829 | -0.513 | 0.0341 | No |
| 39 | 327277 | sec22bb |  |  | 26082 | -0.536 | 0.0425 | No |
| 40 | 100536636 | stx2b |  |  | 26197 | -0.547 | 0.0556 | No |
| 41 | 405846 | stx11a |  |  | 26408 | -0.568 | 0.0663 | No |
| 42 | 567385 | vti1a |  |  | 26783 | -0.607 | 0.0730 | No |
| 43 | 565822 | bnip1a |  |  | 26966 | -0.626 | 0.0863 | No |
| 44 | 394011 | vti1b |  |  | 28249 | -0.778 | 0.0697 | No |
| 45 | 393294 | use1 |  |  | 30434 | -1.467 | 0.0457 | No |
Table: GSEA details
[plain text format]

  

Fig 2: SNAP\_RECEPTOR\_ACTIVITY(GO:0005484)
  
Blue-Pink O' Gram in the Space of the Analyzed GeneSet

  

Fig 3: SNAP\_RECEPTOR\_ACTIVITY(GO:0005484): Random ES distribution
  
Gene set null distribution of ES for
 **SNAP\_RECEPTOR\_ACTIVITY(GO:0005484)**
